# Hypoxia Promotes Dopaminergic Differentiation of Mesenchymal Stem Cells and Shows Benefits for Transplantation in a Rat Model of Parkinson’s Disease

**DOI:** 10.1371/journal.pone.0054296

**Published:** 2013-01-16

**Authors:** Yue Wang, Jian Yang, Haisheng Li, Xuan Wang, Lingling Zhu, Ming Fan, Xiaomin Wang

**Affiliations:** 1 Department of Physiology and Neurobiology, Capital Medical University, Key Laboratory for Neurodegenerative Disease of Education Ministry, Youanmen, Beijing, China; 2 Neuroscience Research Institute, Peking University, Key Laboratory of Neuroscience (PKU), Ministry of Education, Peking University Health Science Center, Beijing, China; 3 Beijing An Ding Hospital, Beijing, China; 4 Department of Brain Protection, Beijing Institute of Basic Medical Sciences, Beijing, China; National Institute of Health, United States of America

## Abstract

Mesenchymal stem cells (MSCs) are multipotent cells capable of differentiating into dopaminergic (DAergic) neurons, which is one of the major cell types damaged in Parkinson’s disease (PD). For this reason, MSCs are considered a potential cell source for PD therapy. It has been proved that hypoxia is involved in the proliferation and differentiation of stem cells. In this study, we investigated the effect of hypoxia on MSC proliferation and DAergic neuronal differentiation. Our results demonstrate that 3% O_2_ treatment can enhance rat MSC proliferation by upregulation of phosphorylated p38 MAPK and subsequent nuclear translocation of hypoxia inducible factor (HIF)-1α. During neural differentiation, 3% O_2_ treatment increases the expression of HIF-1α, phosphorylated ERK and p38 MAPK. These changes are followed by promotion of neurosphere formation and further DAergic neuronal differentiation. Furthermore, we explored the physiological function of hypoxia-induced DAergic neurons from human fetal MSCs by transplanting them into parkinsonian rats. Grafts induced with hypoxia display more survival of DAergic neurons and greater amelioration of behavioral impairments. Altogether, these results suggest that hypoxia can promote MSC proliferation and DAergic neuronal differentiation, and benefit for intrastriatal transplantation. Therefore, this study may provide new perspectives in application of MSCs to clinical PD therapy.

## Introduction

Parkinson’s disease (PD) is a progressive neurodegenerative disorder characterized by the loss of dopaminergic (DAergic) neurons in the substantia nigra [1]. Stem cell-based replacement therapy has been proposed as a promising approach for treating PD. Up to now, several kinds of stem cells have been demonstrated their potential of DAergic neuronal differentiation, including embryonic stem cells (ESCs), neural stem cells (NSCs), induced pluripotent stem (iPS) cells and so on [2], [3], [4], [5]. However, there are certain limitations, such as ethical concerns, tumorigenesis, histocompatibility and inadequate tissue supply.

Since bone marrow mesenchymal stem cells (MSCs) are relatively easy to isolate and expand for autotransplantation, they are considered a potential cell source for cell replacement therapy. In addition, they are multipotent and capable of differentiating into osteocytes, adipocytes, chondrocytes or even neural lineage [6], [7], [8]. It has been demonstrated that MSCs could generate DAergic neurons and show therapeutic potential for PD [9], [10], [11]. However, low *in vivo* survival and DAergic differentiation of MSCs after transplantation has limited their effectiveness and thus clinical application.

Oxygen (O_2_) is an important physiological regulator for cell function. In recent years, the effect of oxygen on the proliferation and differentiation of stem cells has gained more attentions. Several *in vitro* approaches have shown that proliferation and neuronal differentiation, especially DAergic neuronal differentiation, of NSCs are enhanced under hypoxia condition [12], [13], [14]. Verified by neurotransmitter production and electrophysiological activity, DAergic neurons generated in hypoxia displayed much better physiological function compared with those generated in normoxia. In addition, it has been reported that hypoxia could enhance *in vitro* proliferation and *in vivo* survival of transplanted MSCs [15], [16], [17]. Therefore, hypoxia is a promising approach beneficial to cell replacement therapy.

However, molecular mechanisms of how hypoxia promotes cellular proliferation and differentiation are not fully elucidated. As an oxygen-sensitive transcriptional activator, hypoxia- inducible factor (HIF)-1, which plays essential roles in mammalian development, physiology and disease pathogenesis, is supposed to be a primary factor mediating hypoxic responses [18], [19]. HIF-1 consists of two subunits, HIF-1α and HIF-1β. HIF-1β does not respond to changed oxygen concentration. Whereas HIF-1α is rapidly degraded by the ubiquitin-proteosome pathway during normoxia, but under hypoxia, HIF-1α could keep stabilized and translocate from the cytoplasm to the nucleus, leading to the expression of its target genes, such as erythropointin (EPO) and vascular endothelial growth factor (VEGF) [20]. In addition to hypoxia, mitogen-activated protein kinase (MAPK) pathway is also implicated as regulators of HIF-1α [21], [22], [23]. Activation of the extracellular signal-related kinases (ERK)-1/2 MAPK pathway induced the phosphorylation and shift of HIF-1α, and thus promoted its transcriptional activity [22]. Expression of dominant-negative ERK1/2 mutants reduced HIF-1-dependent transcription of the hypoxia-responsive reporter gene [24]. Furthermore, p38 MAPK could be activated by hypoxia in various types of cells [21], [25], and the p38 inhibitor could block hypoxia-mediated proliferation and partially abrogated HIF-1 expression in human pulmonary artery fibroblast [26].

In the present study, we seek to find out the role of hypoxia in the proliferation and neural differentiation of adult rat MSCs (rMSCs) *in vitro*. The mechanism involving HIF-1α and MAPK signaling pathway is also discussed. Moreover, we evaluate the therapeutic efficacy of hypoxia-induced DAergic neurons from human MSCs (hMSCs) after transplantation into the striatum of 6-hydroxydopamine (6-OHDA) lesioned rats. The results aim to provide theoretical and experimental foundations for application of MSCs to clinical PD therapy.

## Materials and Methods

### Ethics Statement

All surgical interventions were carried out in accordance with the Chinese regulations involving animal protection and approved by the Ethics Committee on Animal Care and Usage of Peking University Health Science Center. Permission to use human embryonic tissue was granted by the Ethical Committee of Peking University and with the patient’s written informed consent.

### Animals

Adult Sprague-Dawley (SD) rats (male, 120–160 g; female, 250–350 g) were obtained from the Experimental Animal Centre, Health Science Centre of Peking University. Animals were maintained in a 12-h light/dark cycle in cages with free access to food and water.

### MSCs Isolation and Culture

Bone marrow MSCs were isolated from the femur bone marrow of adult male SD rats or a natural aborted human fetus at 20-weeks gestational age. The procedures were described as follow. Briefly, the bone marrow was aspirated from the femurs aseptically and washed out the fat. Percoll separated fluid (Pharmacia) was added to the cell mass. After centrifugation at 6 000 rpm for 30 min, mist-like cell layer was transferred to the medium consisting of Low Glucose (for rMSCs)/High Glucose (for hMSCs) Dulbecco’s Modified Eagle’s Medium (DMEM-LG/HG, GIBCO BRL), with 10% fetal bovine serum (FBS, Hyclone), 1% glutamine (Sigma-Aldrich) and 1% penicillin (Sigma-Aldrich). Non-adherent cells were removed 24 h later and culture medium was replaced every 2 days.

### Proliferation Assay of MSCs Cultured in Hypoxia

After continuous exposure to different oxygen concentration (21%, 10%, 5%, 3% and 1% O_2_, respectively) *in vitro*, the number of MSCs was assessed by MTT assay. Absorbance was monitored at 570 nm wavelength by an automatic plate reader (BioRad Company, USA). Cell cycle was analyzed by propidium iodide (PI) staining using a FACS Calibur flow cytometer (Becton Dickson). Briefly, cells were collected and washed twice with PBS. Then cells were fixed in 75% cold ethanol over night. After that, cells were treated with RNase A (0.5 mg/ml) for 20 min at 37°C, and stained with 0.05 mg/ml PI (Sigma-Aldrich) at 4°C for 30 min for flow cytometry.

### BrdU Incorporation and Immunostaining

MSCs cultured under 21% O_2_ or 3% O_2_ were labeled with 5 µM 5-bromo-2'-deoxyuridine (BrdU) (Roche Diagnostics) for 48 h to assess mitotic activity. Cells were fixed with cold acetone for 15 min at room temperature (RT). After washing with PBS, cells were denatured with 2 N HCl for 10 min at 37°C. Then cells were incubated with normal goat serum for 1 h at 37°C. Mouse anti-BrdU antibody (Sigma-Aldrich) was added and incubated at 4°C over night. After washing steps, cells were incubated with FITC-conjugated goat anti-mouse IgG for 1 h at 37°C. Images were viewed using a TE2000 Nikon microscopy.

### Neuronal Differentiation of MSCs

After 3–4 passages, rMSCs were digested by 0.25% trypsin/0.02% EDTA and plated as a suspension in neurosphere culture medium, consisting of Neurobasal A, B27, 40 ng/ml basic fibroblast growth factor (bFGF), 20 ng/ml epidermal growth factor (EGF). Primary neurospheres were formed 5–7 days later. To identify the cell types, neurospheres generated under 21% O_2_ or 3% O_2_ were collected and the expression of nestin was detected by immunofluorescence and western blot. For further neuronal differentiation, neurospheres were dissociated by 0.25% trypsin/0.02% EDTA and plated onto poly-L-lysine-coated dishes at appropriate cell density. Cells were induced in differentiation medium (DM) containing Neurobasal A, B27, 20 ng/ml bFGF and 10 µM forskolin. Half of the medium was replaced every 2–3 days. Differentiated cells were examined 10–14 days later for the expression of neuron-specific class III beta-tubulin (TUJ-1), glial fibrillary acidic protein (GFAP) and tyrosine hydroxylase (TH) by immunofluorescence. For neuronal differentiation of hMSCs, cells were plated onto fibronectin-coated dishes at appropriate cell density and transferred to pre-induction medium (DMEM-HG, 20% FBS and 1 mM β-mercaptoethanol) 24 h before neuronal induction. To initiate neuronal differentiation, the pre-induction medium was replaced with neuronal induction medium composed of DMEM-HG and 5 mM β-mercaptoethanol. Differentiated hMSCs were examined 5 h later for the expression of TUJ-1 and TH by immunofluorescence.

### The Effect of HIF-1α and MAPKs on the Proliferation and Differentiation of rMSCs in Hypoxia

To explore the role of HIF-1α and MAPK signaling pathway in the proliferation and differentiation of rMSCs under hypoxia condition, HIF-1α inhibitor echinomycin (5 nM, Sigma-Aldrich), c-jun N-terminal kinase (JNK) inhibitor SP600125 (10 µM, Sigma-Aldrich), ERK inhibitor PD98059 (10 µM, Sigma-Aldrich), and p38 inhibitor SD203580 (10 µM, Sigma-Aldrich) were added into the medium 6 h before hypoxia treatment, respectively. In proliferation assay, expression of proliferating cell nuclear antigen (PCNA) was examined by western blot. In differentiation assay, nestin expression was examined by immunofluorescence; key transcriptional factors involved in the development of DAergic neurons, including *En1*, *En2*, *Nurr1*, *Pitx3* and *Lmx1b* were measured by real-time PCR.

### Immunocytochemistry

Cells were fixed with cold acetone for 15 min at RT and then treated with 0.5% Triton X-100 for 30 min at 37°C. After blocked with 3% normal goat serum, cells were incubated with primary antibodies against nestin (1∶500, Chemicon), TUJ-1 (1∶500, Sigma-Aldrich), GFAP (1∶500, Chemicon) and TH (1∶1 000, Sigma-Aldrich) at 4°C overnight, respectively. Antibody reaction was visualized with FITC/TRITC-conjugated anti-mouse or anti-rabbit secondary antibodies. Images were acquired with a NIKON TE2000 microscopy.

### Western Blot

Cells were lysed with SDS buffer (62.5 mM Tris-HCl, 2% SDS, 10% glycerol, and 50 mM dithiothreitol). Soluble proteins (50–100 µg) were separated by 10% SDS-PAGE and transferred to nitrocellulose membranes (Millipore). Primary antibodies used to probe blots were as follows: mouse monoclonal anti-PCNA (Sigma-Aldrich), mouse monoclonal anti-nestin (Chemicon), rabbit polyclonal anti-Nurr1 (Santa Cruz), mouse monoclonal anti-HIF-1α (Sigma-Aldrich), mouse monoclonal anti-HIF-1β (Sigma-Aldrich), rabbit polyclonal anti-JNK/p-JNK (Cell Signaling), rabbit polyclonal anti-ERK (Cell Signaling), mouse monoclonal anti-p-ERK (Cell Signaling), rabbit polyclonal anti-p38/p-p38 (Cell Signaling), mouse monoclonal anti-actin (Sigma-Aldrich), and mouse monoclonal anti-GAPDH (Sigma-Aldrich). Membranes were then incubated with IRDye 800 or IRDye 700 conjugated anti-mouse or anti-rabbit secondary antibodies (Rockland Immunochemicals). Signals were visualized by Odyssey Infrared Imaging System (LI-COR Biosciences).

### Quantitative Real-time PCR

Total RNA was extracted from cells using TRIzol reagent (Invitrogen) and reversely transcripted for cDNA synthesis with SuperScript III cDNA synthesis kit (Invitrgen). Real-time PCR was performed with the Brilliant SYBR Green QPCR Master Mix kit using Mx3000P Sequence Detection system (Stratagene). GAPDH was used as an internal control to quantify and normalize the results. Primers for amplification of target cDNA were provided in Table S1. The data was analyzed using MxPro QPCR software.

### Establishment of Parkinsonian Rat Model and Cell Transplantation

Parkinsonian rat model establishment and cell transplantation were performed as described previously [27]. Briefly, total 60 adult female SD rats (8 weeks of age, 250–350 g) were unilaterally injected with 8 µg 6-OHDA (Sigma-Aldrich) into the right medial forebrain bundle at the coordinates as follows: AP = −4.3 mm, ML = −1.5 mm, DV = −7.5 mm [28]. Four weeks later, rotational behavior of rats were triggered by apomorphine (0.5 mg/kg injected subcutaneously, Sigma-Aldrich) challenge. Rotational scores were collected for 30 min in a computer-assisted rotometer system (Rota-count 8, Columbus instruments, Columbus). Only rats exhibiting five or more net contralateral rotations/min were selected for further study. Successful parkinsonian rats were randomly classified into four groups transplanted with basic medium (vehicle control), intact hMSCs, i-hMSCs and hi-hMSCs, respectively. Cells for transplantation were suspended in basic medium without any cytokine at a density of 100 000/µl. Then the cell suspension was injected into ipsilateral *striatum* of rats at two points (2.5 µl for each site, AP = +1.0 mm, ML = −3.0 mm, DV =  −4.5/−5.0 mm). Apomorphine-induced rotational behavior was examined at 2, 4, 6 and 8 weeks after transplantation, respectively.

### Measurement of DA and its Metabolites

Rats were sacrificed at the eighth week after transplantation by decapitation. The striatum was immediately weighed and then homogenized in 0.1 M ice-cold perchloric acid. DA and its metabolites, dihydroxyphenylacetic acid (DOPAC) and homovanillic acid (HVA), were measured by HPLC with electrochemical detection. Results were presented as the ratio of the content (lesioned side *vs*. unlesioned side).

### Assessment of Cell Transplantation

Eight weeks after transplantation, rats were deeply anesthetized and perfused for immunohistochemical analysis. In brief, brain tissues were sliced into 30 µm-thick sections on a cryostat. Every twelfth section of the *graft* was collected for further processing. After blocked with 3% normal goat serum, brain slices were incubated with mouse anti- human nuclei (HuN, 1∶200, Chemicon) or mouse anti-TH (1∶2 000, Sigma-Aldrich) overnight at 4°C. The antibody was detected using an ABC kit (Vector Laboratories) with 3,3′-diaminobenzidine (DAB). Cell number was estimated by counting the number of HuN^+^ or TH^+^ cells through the graft. For immunofluorescent staining, sections were double-stained with HuN and rabbit anti-Neurofilament (NF)-M (1∶200, Chemicon) or GFAP (1∶200, Chemicon), respectively. Images were acquired with a confocal laser scanning microscopy (CLSM, Leica, Germany). The percentage of double-positive cells was estimated roughly by counting the double-stained cell number in 100 randomly selected HuN^+^ cells. The unlesioned side was used as the control relative to the lesioned side. All sections were evaluated in double-blinded manner.

### Statistical Analysis

All data were presented as means ± SEM. Statistical significance was assessed with Student’s *t*-test or one-way analysis of variance (ANOVA) using Prism 4.0 software (GraphPad Software). *P* value less than 0.05 was considered as statistically significant.

## Results

### The Effect of Physiological Hypoxia on the Proliferation of rMSCs

RMSCs were isolated from the femur bone marrow of adult male SD rats. Phenotypic characteristics were determined by flow cytometry analysis. Approximately 88% of the rMSCs were found to express CD44 and CD71, respectively, but never express CD34 and CD45, the markers for hematopoietic stem cells (HSCs) [29] (Fig. S1A).

**Figure 1:**
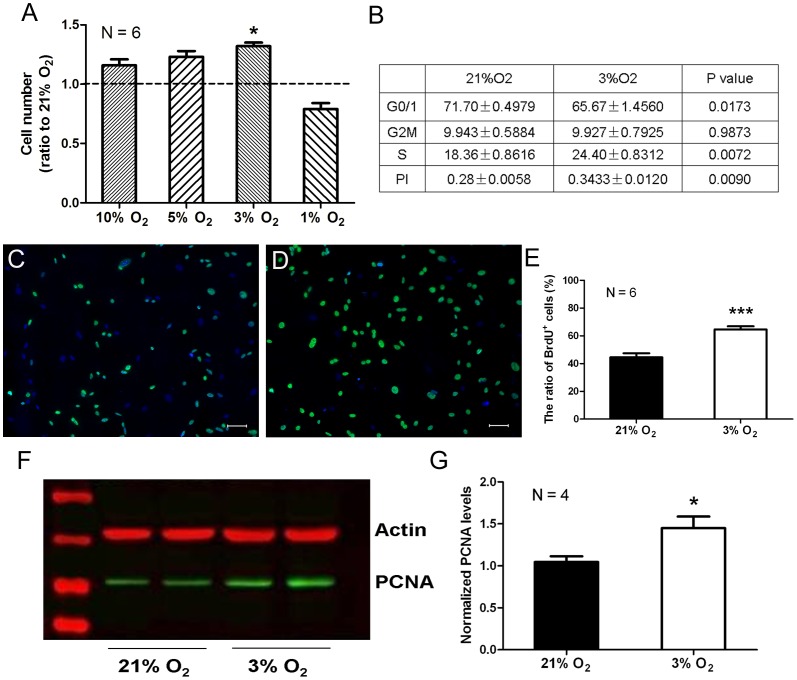
Physiological hypoxia promotes rMSC proliferation. (A) Cell counting of rMSCs cultured under different oxygen concentration. Cell number is normalized to that of 21% O_2_ (dashed line). The data are presented as means ± SEM of 6 individual wells. (B) Cell cycle analysis by FACS. The data are presented as a percentage of 3 individual wells. PI (proliferous index) = [(S+G2M) ÷ (G0/1+ S+G2M)] × 100%. (C, D) BrdU incorporation assay for rMSCs cultured under 21% O_2_ (C) or 3% O_2_ (D). Scale bar  = 50 µm. (E) Quantitative analysis of BrdU incorporation in C and D. The data are presented as means ± SEM of 6 individual wells. (F) Western blot analysis of PCNA expression in rMSCs cultured under 21% O_2_ and 3% O_2_, respectively. The graph presented is representative of 4 independent experiments with similar results. (G) Quantitative analysis of PCNA in F. *P<0.05, ***P<0.001.

To explore the impact of hypoxia on the proliferation, rMSCs were exposed to normoxic (21% O_2_) and different hypoxic conditions (10%, 5%, 3% and 1% O_2_, respectively) in cell culture for 3 days. Cell number was evaluated by hemacytometer counts. The result showed that the number of rMSCs in 3% O_2_ group was significantly higher than that in 21% O_2_ group (Fig. 1A). Thus, 3% O_2_ was considered as the most suitable hypoxic condition for rMSC proliferation in the present study. Further, cell cycle analysis by PI staining demonstrated that the ratio of S-phase rMSCs in 3% O_2_ group was 33% higher than that in 21% O_2_ group (Fig. 1B), indicating that 3% O_2_ could evoke more active DNA synthesis. This hypothesis was validated by increased BrdU incorporation and PCNA expression. BrdU can be incorporated into the newly synthesized DNA during the S phase of the cell cycle. Immunocytochemical results showed that percentage of BrdU^+^ cells in 3% O_2_ group rose to 1.45 times of that in 21% O_2_ group (Fig. 1C–1E). PCNA acts as a processivity factor for DNA polymerase delta and was originally identified as an antigen expressed in the nuclei of cells during the S phase of the cell cycle. The protein level of PCNA in 3% O_2_ group was approximately 40% more than that in 21% O_2_ group (Fig. 1F and 1G). In addition, phenotypic characteristics of rMSCs were not changed by hypoxia treatment (Fig. S1B). These findings demonstrate that hypoxia can promote the proliferation of rMSCs *in vitro*.

### HIF-1α and p38 are Involved in the Proliferation-promoting Effect of 3% O_2_ on rMSCs

**Figure 2:**
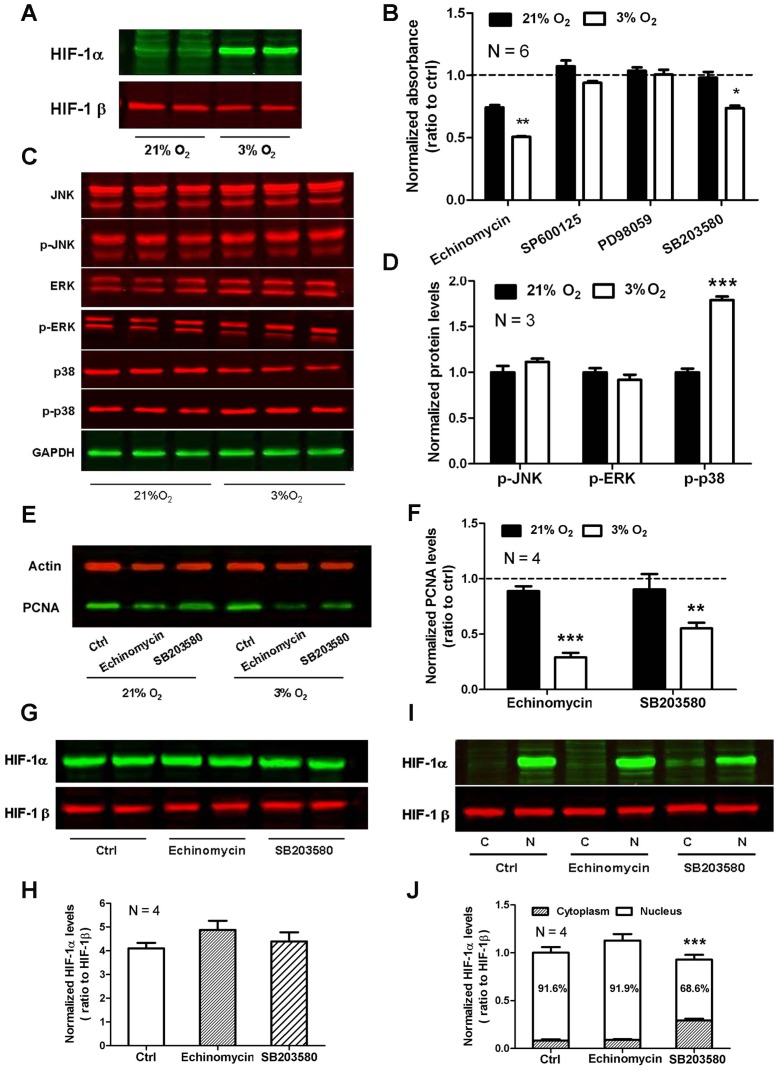
HIF-1α and p38 are involved in hypoxia-induced rMSC proliferation. (A) HIF-1α is stably expressed in rMSCs cultured under 3% O_2_. (B) MTT array for the effect of HIF-1α and MAPKs inhibitors on cell viability of rMSCs cultured under 21% O_2_ or 3% O_2_. The data are presented as means ± SEM of 6 individual wells. (C) Western blot analysis of phosphorylated JNK, ERK and p38 in rMSCs cultured under 21% O_2_ or 3% O_2_. Total JNK, ERK and p38 were used as their respective internal control. The graph presented is representative of 3 independent experiments with similar results. (D) Quantitative analysis of phosphorylated JNK, ERK and p38 in C. (E) Western blot analysis of PCNA in rMSCs after treatment with HIF-1α inhibitor echinomycin or p38 inhibitor SB203580. The graph presented is representative of 4 independent experiments with similar results. (F) Quantitative analysis of PCNA in E. (G) Western blot analysis of total HIF-1α in rMSCs under 3% O_2_ after treatment with echinomycin or SB203580. HIF-1β was used as an internal control. The graph presented is representative of 4 independent experiments with similar results. (H) Quantitative analysis of HIF-1α in G. (I) Western blot analysis of cytoplasmic and nuclear HIF-1α in rMSCs under 3% O_2_ after treatment with echinomycin or SB203580. The graph presented is representative of 4 independent experiments with similar results. (J) Quantitative analysis of cytoplasmic and nuclear HIF-1α in I. Data represent mean ± SEM, *P<0.05, **P<0.01, ***P<0.001.

HIF-1α is the hypoxically responsive component of HIF-1 [30]. Under normoxic condition, two proline residues of HIF-1α can readily be hydroxylated. Subsequently, HIF-1α is rapidly ubiquitinated and degraded by proteasome [31]. Since prolyl hydroxylation utilizes oxygen as a cosubstrate, it can be inhibited under hypoxic conditions, which keeps HIF-1α stabilized. In the present study, protein level of HIF-1α was stably expressed in 3% O_2_ group (Fig. 2A). When pre-treated with echinomycin, a small-molecule inhibitor of HIF-1α DNA-binding activity, 3% O_2_-induced proliferative effect and upregulation of PCNA were abolished (Fig. 2B, 2E and 2F), suggesting HIF-1α may participate in the hypoxia-induced proliferation of rMSCs.

To investigate the possible involvement of MAPK signaling pathway in the proliferative effects of hypoxia and the relevance with HIF-1α, the expression of MAPKs was measured using western blot. As shown in Fig. 2C, phosphorylated p38 was enhanced remarkably in hypoxia condition, while phosphorylated ERK and JNK showed no significant change. When respectively pre-treated with JNK inhibitor SP600125, ERK inhibitor PD98059 and p38 inhibitor SB203580, an obvious decrease in cell viability was only detected in SB203580-treated 3% O_2_ group (Fig. 2B). Meanwhile, these three inhibitors mentioned above triggered little cell death as shown by double staining with Hoechst 33342 and PI (data not shown). In addition, SB203580 could also reverse the upregulation of PCNA in 3% O_2_ group (Fig. 2E). The above results indicate that p38 signaling pathway is possibly involved in the effect of hypoxia on rMSC proliferation.

As described above, both HIF-1α and p38 are involved in the promotion of rMSC proliferation under hypoxia condition. We next explored the relationship between HIF-1α and p38 using western blot. The results revealed that neither echinomycin nor SB203580 affected the total protein level of HIF-1α under 3% O_2_ (Fig. 2G and 2H). However, when cytoplasm and nucleus were separated, the ratio of nuclear HIF-1α was diminished to 75% of control group after treatment with SB203580, but not echinomycin (Fig. 2I and 2J). Therefore, hypoxia increases HIF-1α nuclear translocation partially depended on the upregulation of phosphorylated p38.

### The Promotion of Physiological Hypoxia on the Neural Differentiation of rMSCs

**Figure 3:**
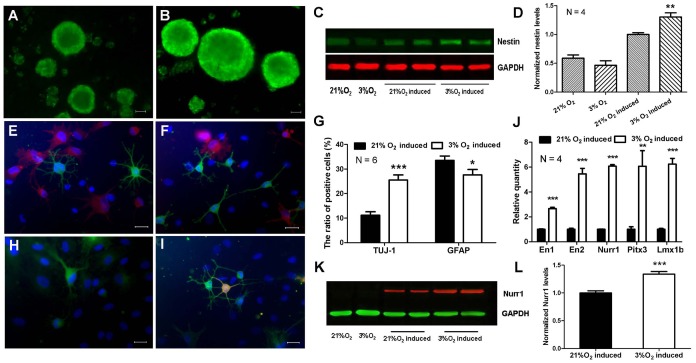
Physiological hypoxia promotes neural differentiation of rMSCs. (A, B) Immunostaining of nestin in rMSC-derived neurospheres generated under 21% O_2_ (A) or 3% O_2_ (B). Scale bar  = 100 µm. (C) Western blot analysis of nestin in rMSC-derived neurospheres generated under 21% O_2_ or 3% O_2_. The graph presented is representative of 4 independent experiments with similar results. (D) Quantitative analysis of nestin in C. (E, F) Double immunostaining of TUJ-1 (green) and GFAP (red) in differentiated neurospheres generated under 21% O_2_ (E) or 3% O_2_ (F). Scale bar  = 20 µm. (G) The percentage of TUJ-1^+^ or GFAP^+^ cells in E and F. The data are presented as means ± SEM of 6 individual wells. (H, I) Double immunostaining of TUJ-1 (green) and TH (red) in differentiated neurospheres generated under 21% O_2_ (H) or 3% O_2_ (I). Scale bar  = 20 µm. (J) The transcription levels of *En1*, *En2*, *Nurr1*, *Pitx3* and *Lmx1b* in rMSC-derived neurospheres generated under 21% O_2_ or 3% O_2_ were detected by real-time PCR. The data presented are normalized to that in 21% O_2_, respectively (*n*  = 4). (K) Western blot analysis of Nurr1 in rMSC-derived neurospheres generated under 21% O_2_ or 3% O_2_. The graph presented is representative of 4 independent experiments with similar results. (L) Quantitative analysis of Nurr1 in K. Data represent mean ± SEM, *P<0.05, **P<0.01, ***P<0.001.

To explore the impact of hypoxia on the neural differentiation, we plated rMSCs as a suspension in neurosphere culture medium under different oxygen concentration (21%, 10%, 5%, 3% and 1% O_2_, respectively). Neurosphere-like cell clusters formed in 3% O_2_ group was the largest among these groups after 2 days (Fig. S2A). Moreover, rMSCs induced under 3% O_2_ aggregated much faster than those induced under 21% O_2_ (Fig. S2B). Immunocytochemical results showed that these neurosphere-like cell clusters generated under 3% or 21% O_2_ were both positive for nestin, a marker of NSCs (Fig. 3A and 3B). Western blot demonstrated that expression of nestin in 3% O_2_ group was much higher than that in 21% O_2_ group after induction, although rMSCs without induction also expressed nestin weakly (Fig. 3C and 3D). In addition, the expression of surface antigens CD44 and CD71 was remarkably decreased after induction, especially in 3% O_2_ group (Fig. S3). These results imply that hypoxia can promote neural conversion of rMSCs.

For neuronal differentiation, both neurosphere-like cell clusters generated in 3% O_2_ group and 21% O_2_ group were dissociated and plated on poly-L-lysine coated dishes or coverslips in DM under normoxia. After 10-14 days, polypolar and flat cobble-like cells were observed. Immunocytochemistry revealed that differentiated cells expressed neuronal marker TUJ-1 or astroglia marker GFAP (Fig. 3E and 3F), which confirmed the multi-differentiation property of the neurospheres. Furthermore, the ratio of TUJ-1^+^ neurons differentiated from 3% O_2_-induced neurospheres was 1.27 times more than that from 21% O_2_, while 3% O_2_-induced neurospheres differentiated into fewer astrocytes (Fig. 3G). Among the TUJ-1^+^ neurons, glutamic acid decarboxylase (GAD)-67 or choline acetyltransferase (ChAT)-positive cells were observed in both groups, and we found no significant percentage difference within the two groups, respectively (Fig. S4).

However, TH^+^ neurons could only be detected in 3% O_2_-induced neurospheres (7.32±1.88%) (Fig. 3H and 3I). TH is the rate-limiting enzyme in the production of DA and considered a basic marker for DAergic neurons. To confirm their potential of DAergic neuronal differentiation, real-time PCR was used to examine several important genes involved in the development of DAergic neurons *in vivo*, including *En1*, *En2*, *Nurr1*, *Pitx3* and *Lmx1b*. As shown in Fig. 3J, mRNA for these genes was significantly elevated in 3% O_2_-induced neurospheres. Western blot further demonstrated a higher expression of Nurr1 in 3% O_2_-induced neurospheres (Fig. 3K and 3L). These results indicate that neurospheres induced under hypoxia condition prefer to differentiate into neurons, typically DAergic neurons.

### HIF-1α, ERK and p38 are Involved in the Neural Differentiation-promoting Effect of 3% O_2_ on rMSCs

**Figure 4:**
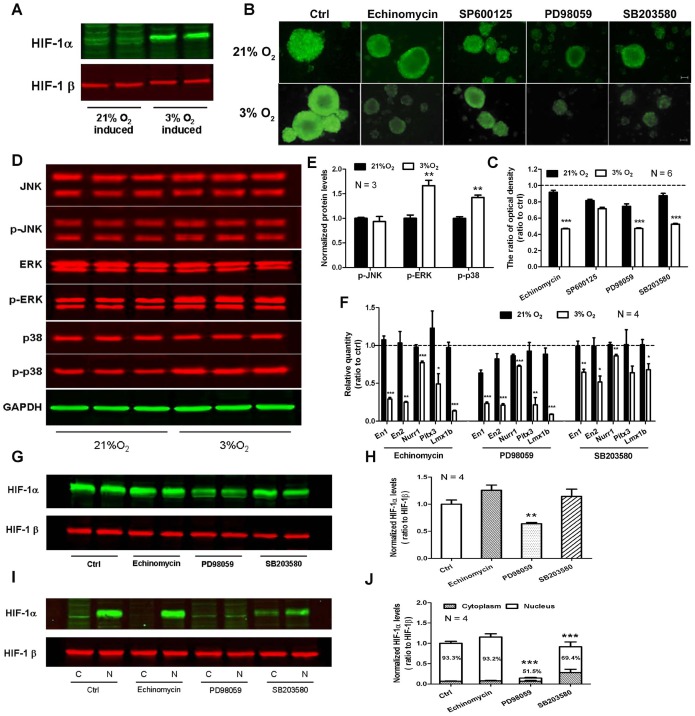
HIF-1α, ERK and p38 are involved in hypoxia-induced neural differentiation of rMSCs. (A) HIF-1α is stably expressed in 3% O_2_-induced neurospheres. (B) Immunostaining of nestin in rMSC-derived neurospheres after respective treatment with HIF-1α inhibitor echinomycin, JNK inhibitor SP600125, ERK inhibitor PD98059 and p38 inhibitor SB203580. Scale bar  = 100 µm. (C) Quantitative analysis of optical density of nestin expression in B by densitometry. The data are presented as means ± SEM of 6 individual wells. (D) Western blot analysis of phosphorylated JNK, ERK and p38 in rMSC-derived neurospheres. The graph presented is representative of 3 independent experiments with similar results. (E) Quantitative analysis of phosphorylated JNK, ERK and p38 in D. (F) The effect of inhibitors on mRNA of *En1*, *En2*, *Nurr1*, *Pitx3* and *Lmx1b* in rMSC-derived neurospheres. The data presented are normalized to their respective control (*n*  = 4). (G) Western blot analysis of total HIF-1α in 3% O_2_-induced neurospheres after respective treatment with echinomycin, PD98059 and SB203580. HIF-1β was used as an internal control. The graph presented is representative of 4 independent experiments with similar results. (H) Quantitative analysis of HIF-1α in G. (I) Western blot analysis of cytoplasmic and nuclear HIF-1α in 3% O_2_-induced neurospheres after respective treatment with echinomycin, PD98059 and SB203580. The graph presented is representative of 4 independent experiments with similar results. (J) Quantitative analysis of cytoplasmic and nuclear HIF-1α in I. Data represent mean ± SEM, *P<0.05, **P<0.01, ***P<0.001.

Neurospheres generated under 3% O_2_ could stably expressed HIF-1α (Fig. 4A). When pre-treated with echinomycin, neurospheres became much smaller (Fig. 4B and 4C), suggesting that HIF-1α may play a role in neural differentiation of rMSCs under hypoxia condition. As demonstrated by western blot, phosphorylation of ERK and p38 was significantly enhanced, but phosphorylated JNK was not (Fig. 4D and 4E). When respectively pre-treated with their inhibitors, PD98059 and SB203580 strikingly restrained neurosphere formation in 3% O_2_, as shown by decreased nestin expression detected by immunocytochemistry (Fig. 4B). In addition, transcriptional levels of *En1*, *En2*, *Nurr1*, *Pitx3* and *Lmx1b* were also down-regulated after treatment with echinomycin, PD98059 and SB203580, respectively (Fig. 4F). Thus, HIF-1α, ERK and p38 may be involved in the neural differentiation-promoting effect of 3% O_2_ on rMSCs.

To explore the interaction between HIF-1α and ERK or p38, western blot was used to analyze the expression of HIF-1α after respective treatment with echinomycin, PD98059 and SB203580. Results showed that PD98059 could remarkably reduce HIF-1α expression, while echinomycin and SB203580 exerted no effect (Fig. 4G and 4H), suggesting that upregulation of phosphorylated ERK is closely related to enhanced expression of HIF-1α during neural differentiation of rMSCs in hypoxia. Furthermore, when cytoplasm and nucleus were separated, the ratio of HIF-1α in nucleus was diminished to about 55% and 74% of control group after PD98059 and SB203580 treatment, respectively (Fig. 4I and 4J). Based on these results, it can be concluded that, during neural differentiation of rMSCs in hypoxia, upregulation of phosphorylated ERK and p38 could promote the translocation of HIF-1α from cytoplasm to nucleus, and that ERK signaling pathway may be involved in the regulation of HIF-1α expression, thereby demonstrating a neural differentiation-promoting effect of rMSCs.

### Transplantation and Behavioral Recovery of Parkinsonian Rats

As described above, although hypoxia can trigger more DAergic differentiation from rMSCs *in vitro*, the physiological function of them needs further *in vivo* investigations. Thus, we explored the effect of hypoxia-induced DAergic neurons from MSCs after transplantation into parkinsonian rats. Here, we used hMSCs isolated from the femur bone marrow of a natural aborted human fetus, aiming at better simulation of clinical condition.

**Figure 5:**
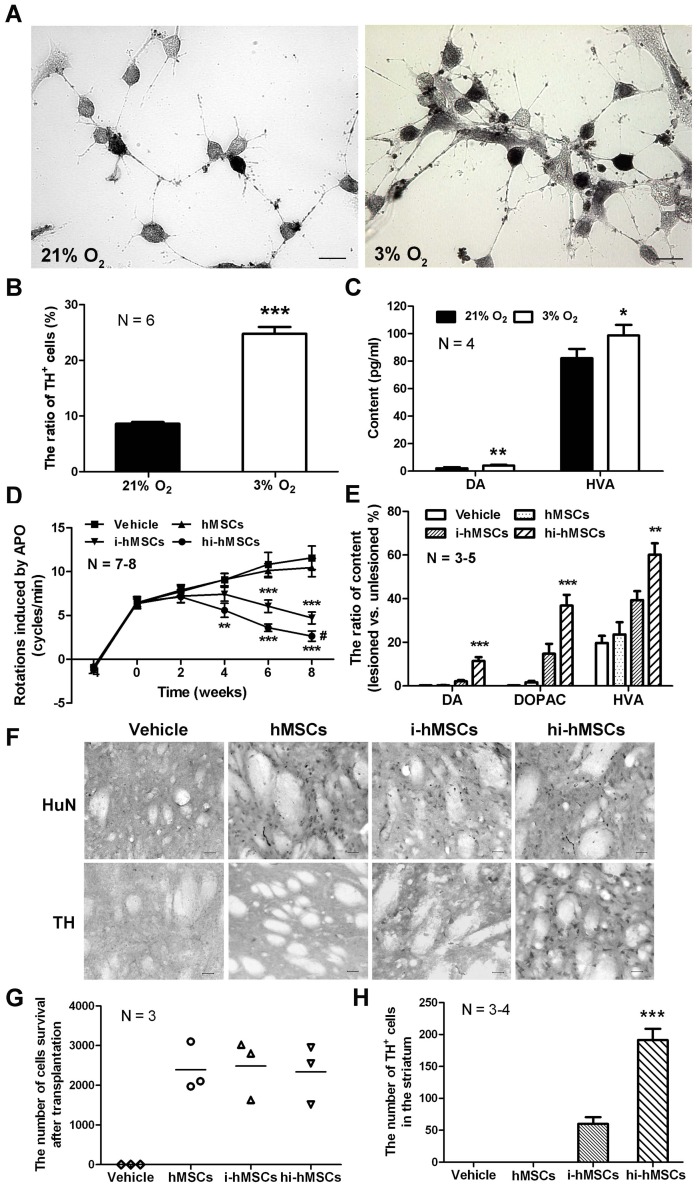
Transplantation and behavioral recovery of parkinsonian rats. (A) TH immunohistochemistry of differentiated hMSCs induced by β-mercaptoethanol under 21% O_2_ or 3% O_2_. Scale bar  = 20 µm. (B) The percentage of TH^+^ cells in A. The data are presented as means ± SEM of 6 individual wells. (C) HPLC detection of DA and HVA content in differentiatiated hMSCs induced under 21% O_2_ or 3% O_2_. The data are presented as means ± SEM of 4 individual wells. (D) Apomorphine-induced rotation from 4 weeks before transplantation to the eighth week after transplantation. *n*  = 7-8 each group. ^#^ *P*<0.05, *versus* the rotation at 0 week in the same group. (E) The relative striatal content of DA and its metabolites, DOPAC and HVA, after transplantation. *n*  = 3-5 each group. (F) Cells survival after transplantation were stained using anti-HuN antibody or anti-TH antibody. (G) Cell count of HuN^+^ cells in F. *n*  = 3 each group. (H) Cell count of TH^+^ cells in F. *n*  = 3-4 each group. Data represent mean ± SEM, *P<0.05, **P<0.01, ***P<0.001.

HMSCs can directly differentiate into DAergic neurons after induction with β-mercaptoethanol [7]. As shown in Fig. 5A, more TH^+^ DAergic neurons were detected in 3% O_2_ group than that in 21% O_2_ group after differentiation (24.8±1.2% *vs*. 8.6±0.3%, Fig. 5B). Similar results were also found by flow cytometry analysis (Fig. S5). To characterize the function of differentiated DAergic neurons from hMSCs cultured *in vitro*, the capacity to synthesize and release DA was assessed by HPLC. The result showed that the level of DA and its metabolite HVA were significantly increased in 3% O_2_ group compared with those in 21% O_2_ group (DA, 4.10±0.54 pg/ml *vs*. 2.019±0.78 pg/ml; HVA, 98.68±7.69 pg/ml *vs*. 82.18±9.65 pg/ml) (Fig. 5C).

Parkinsonian rat models were established by unilateral injection with 6-OHDA into the right medial forebrain bundle (Fig. S6). Intact hMSCs, normoxia-induced hMSCs (i-hMSCs) and hypoxia-induced hMSCs (hi-hMSCs) with equal quantities were transplanted into the striatum on the lesioned side of model rats 4 weeks after 6-OHDA injection. In the 6-OHDA lesioned side of the brain, most of the DAergic neurons in the substantia nigra and their fibers in the striatum were depleted (data not shown), resulting in an abnormal rotation upon apomorphine challenge. Rats transplanted with hi-hMSCs showed significantly improved rotational behavior compared with those transplanted with vehicle control when injected with apomorphine from the fourth weeks after transplantation. This behavioral recovery lasted for up to the eighth week throughout the observation period. In the group transplanted with i-hMSCs, rats also showed better rotational behavior relative to control, from the sixth week after transplantation. In contrast, transplantation with intact hMSCs revealed no effect (Fig. 5D).

The striatal content of DA and its metabolites, DOPAC and HVA, was measured by HPLC. To avoid the individual variances, the result was represented as the ratio of the content in lesioned side *versus* unlesioned side. We found that DA, DOPAC and HVA were all significantly elevated in hi-hMSCs group compared with hMSCs group or i-hMSCs group, respectively (Fig. 5E).

The number of survived cells after transplantation was counted using anti-HuN antibody by immunohistochemistry. There were no significant differences of cell number among the three groups (Fig. 5F and 5G). TH immunohistochemistry was used to clarify the survival of DAergic neurons after transplantation. Normally, there are no DAergic neuronal somas existing in the striatum. Thus, the TH^+^ cell bodies observed in the striatum should be derived from the grafted cells. The number of TH^+^ cells in hi-hMSCs group was two times more than that in i-hMSCs group, while none could be detected in intact hMSCs group or vehicle control group (Fig. 5F and 5H). Moreover, higher percentages of NF-M^+^ or GFAP^+^ cells were found among the HuN^+^ cells in hi-hMSCs group (Fig. S7).

These data indicate that hMSCs possess better differentiation ability to DAergic neurons after induction with β-mercaptoethanol *in vitro* under hypoxia condition. After transplantation into the striatum of parkinsonian rats, grafts induced with hypoxia display more surviving DAergic neurons, significantly elevated DA content in the striatum and great amelioration of behavioral impairments.

## Discussion

In the present study, our results support the following conclusions. First, 3% O_2_ promotes rMSC proliferation by the upregulation of HIF-1α and phosphorylated p38. The hypoxic environment increases HIF-1α nuclear accumulation partially depended on the upregulation of phosphorylated p38. Second, 3% O_2_ promotes neural conversion of rMSCs, as evidenced by more neurosphere formation and neuronal differentiation, typically DAergic neurons. This response is mediated by ERK-related HIF-1α expression and ERK/p38-dependent HIF-1α nuclear translocation. Finally, we suggest that 3% O_2_ can raise the differentiation ratio of hMSCs to DAergic neurons *in vitro*, and these cells better ameliorate behavioral impairment after transplantation into the striatum of parkinsonian rats, possibly related to more surviving DAergic neurons and significantly elevated DA content. Thus, hypoxia promotes DAergic neuronal differentiation of MSCs and leads to an enrichment of differentiated DAergic neurons after intrastriatal transplantation that can be used in stem cell therapy of PD.

Under normal physiological conditions, mean tissue O_2_ concentration is 3%, which is much lower than atmospheric oxygen level [12]. Correspondingly, virtually all stem cells, including MSCs, proliferate better in physiologic oxygen environments [15], [32], [33]. Similarly, we also found that physiological hypoxia (3% O_2_) most significantly increased cell number of rMSCs, whereas lower oxygen concentration (1% O_2_) was deleterious. Further analysis demonstrated that 3% O_2_ enhanced the ratio of S-phase rMSCs, BudU incorporation and PCNA expression.

The MAPK signaling pathway is widely involved in various cellular functions, including growth, differentiation, inflammation and apoptosis. In mammals, there are three major MAPK pathways: ERK, JNK, and p38 MAPK. They can be activated by hypoxia and may play an important role in the proliferation-promoting response to hypoxia [26], [34], [35], [36], [37]. We observed a remarkable increase of phosphorylated p38 level under hypoxic conditions as well as HIF-1α. Hypoxia-induced proliferation of rMSCs was inhibited by pretreatment with HIF-1α inhibitor echinomycin or p38 inhibitor SB203580, but not JNK inhibitor SP600125 or ERK inhibitor PD98059. Several lines of evidence have shown that p38 activation is related to HIF-1α expression in hypoxia [26], [36], [38]. We examined the link between p38 and HIF-1α. Presently, inhibition of p38 did not affect the total protein level of HIF-1α, but partially blocked HIF-1α nuclear translocation. Our results suggest the proliferation-promoting effect of hypoxia partially depends on p38-mediated HIF-1α nuclear translocation.

Hypoxia has been implicated in promoting DAergic neuronal differentiation of ESCs and NSCs through activation of HIF-1α [13], [39], [40], [41]. It has also been shown that the hypoxia-mimetic agent CoCl_2_ induced neuronal differentiation of MSCs through HIF-1α activation and cell-cycle arrest, and that Rho kinase (ROCK) inhibition potentiated CoCl_2_-induced MSC differentiation especially into DAergic neurons [42], [43]. However, the effect of hypoxia on DAergic neuronal differentiation of MSCs remains unclear. In the present study, we found hypoxia promoted neurosphere formation of rMSCs using a general neurosphere protocol [44], [45], [46], [47]. When cultured in DM under normoxia, these hypoxia-induced neurospheres could differentiate into more DAergic neurons, as evidenced by the increased ratio of TH^+^ cells and mRNA levels of *En1*, *En2*, *Nurr1*, *Pitx3* and *Lmx1b*. Similar results were also observed in hMSCs. Taken together, these results suggest that hypoxia promotes DAergic neuronal differentiation of MSCs.

MAPK pathway is also reported to participate in the differentiation-promoting effect of hypoxia [48], [49]. Here, we found HIF-1α, ERK and p38 were significantly activated during neurosphere formation of rMSCs under hypoxia, but not JNK. Inhibition of HIF-1α, ERK or p38 strikingly restrained both neurosphere formation and subsequent differentiation to DAergic neurons of rMSCs. Additionally, inhibition of either ERK or p38 reduced nuclear HIF-1α level, and inhibition of ERK also decreased the total HIF-1α expression. It has been reported that HIF-1α regulates TH gene expression through binding to its hypoxia-responsive element (HRE) in the promoter [20], [50]. Therefore, the effect of hypoxia on DAergic neuronal differentiation of MSCs may be mediated by upregulation of phosphorylated ERK and p38, and subsequent activation of HIF-1α. More importantly, we found distinct involvement of MAPKs in hypoxia-induced HIF-1α activation during MSC proliferation and differentiation, although detailed underlying mechanism requires a further investigation.

For clinical treatment of PD, human-derived MSCs possesses more advantages than those from animals [51]. In our work, we have succeeded in obtaining a higher ratio of DAergic neurons from hMSCs under hypoxia environment. In order to validate their physiological function, we compared among the efficacy of hi-hMSCs, i-hMSCs and intact hMSC after transplanting them into parkinsonian rats. The present results show that intrastriatal transplantation of hi-hMSCs can more efficaciously ameliorate behavioral deficits of parkinsonian rats when compared with that of i-hMSCs or intact hMSC. And the behavioral improvement is accompanied by more surviving DAergic neurons and higher content of DA and its metabolites, DOPAC and HVA, in the striatum. These results indicate that the behavioral recovery may benefit from the elevated level and usage effectiveness of DA produced by hi-hMSCs. Previous studies have shown hypoxia-preconditioned cells displayed reduced cell death and apoptosis after transplantation, and therefore promote their therapeutic potential [16], [17], [52], [53]. However, we did not find more surviving cells in the hi-hMSCs group, and it might be attributed to differential composition of the cell population (differentiated and undifferentiated cells) among the three groups and a long time interval between exposure to normoxia and transplantation.

In conclusion, the present study reveals that hypoxia promotes MSC proliferation and DAergic neuronal differentiation, and benefits for intrastriatal transplantation. Moreover, this work provides evidence for a crosstalk between HIF-1α and MAPK pathway in response to hypoxia. Therefore, this study should help the development of cell replacement therapy for PD and provide new perspectives in clinical application of MSCs.

## Supporting Information

Figure S1 **Flow cytometry analysis of phenotypic characteristics of rMSCs cultured under 21% O_2_ or 3% O_2_ for 3 days.** (A) Phenotypic characteristics of rMSCs cultured under 21% O_2_. (B) Phenotypic characteristics of rMSCs cultured under 3% O_2_.
(JPG)

Figure S2 **3% O_2_ promotes the formation of neurosphere-like cell clusters from rMSCs.** (A) Phase micrographs of rMSCs induced with neurosphere culture medium under different oxygen concentration at day 2. Scale bar  = 100 µm. (B) Phase micrographs of rMSCs induced with neurosphere culture medium at different time points under 21% O_2_ or 3% O_2_. Scale bar  = 100 µm.
(JPG)

Figure S3 **Flow cytometry analysis of phenotypic characteristics of rMSCs induced with neurosphere culture medium under 21% O_2_ or 3% O_2_ for 3 days.** The expression of surface antigens CD44 and CD71 was remarkably decreased after induction, especially in 3% O_2_ group.
(JPG)

Figure S4 **Double immunostaining of GAD67/TUJ-1 or ChAT/TUJ-1 in differentiated neurospheres.** (A, C) Double immunostaining of GAD67 (red) and TUJ-1 (green). (B, D) Double immunostaining of ChAT (red) and TUJ-1 (green). (A, B) Double immunostaining for differentiated neurospheres generated under 21% O_2_. (C, D) Double immunostaining for differentiated neurospheres generated under 3% O_2_. (E) The percentage of GAD67^+^ or ChAT^+^ cells in TUJ-1^+^ cells in A–D. Scale bar  = 20 µm for A–D.
(JPG)

Figure S5 **Flow cytometry analysis of TH^+^ cells in differentiated hMSCs.** (A) Isotype control. (B) hMSCs differentiated under 21% O_2_. (C) hMSCs differentiated under 3% O_2_.
(JPG)

Figure S6 **Establishment of parkinsonian rat model by unilateral injection with 6-OHDA into right medial forebrain bundle.** (A, B) Immunohistochemistry staining of TH in the substantia nigra of brain sections from saline (A) or 6-OHDA (B) injected rats. (C) The number of apomorphine-induced rotation 4 weeks after 6-OHDA injection. (D) The relative striatal content of DA and its metabolites, DOPAC and HVA (lesioned side *vs*. unlesioned side). Data represent mean ± SEM, ***P<0.001.
(JPG)

Figure S7 **Double immunostaining of NF-M/HuN or GFAP/HuN in the striatum after transplantation.** (A–C) Double immunostaining of NF-M (red) and HuN (green). (D–F) Double immunostaining of GFAP (red) and HuN (green). (A, D) PD model rats transplanted with hMSCs. (B, E) PD model rats transplanted with i-hMSCs. (C, F) PD model rats transplanted with hi-hMSCs. (G) The percentage of NF-M^+^ cells in HuN^+^ cells in A–F. (H) The percentage of GFAP^+^ cells in HuN^+^ cells in A–F. Scale bar = 8 µm for A–E. Scale bar  = 20 µm for F. Data represent mean ± SEM, *P<0.05, **P<0.01.
(JPG)

Table S1 **Primer sequences for real-time PCR.**
(DOC)
