# Supplementary figures and images for: Hypoxia Promotes Dopaminergic Differentiation of Mesenchymal Stem Cells and Shows Benefits for Transplantation in a Rat Model of Parkinson’s Disease
Source: PLoS One. 2013 Jan 16;8(1):e54296. doi: 10.1371/journal.pone.0054296 (PMC3546985; doi:10.1371/journal.pone.0054296)

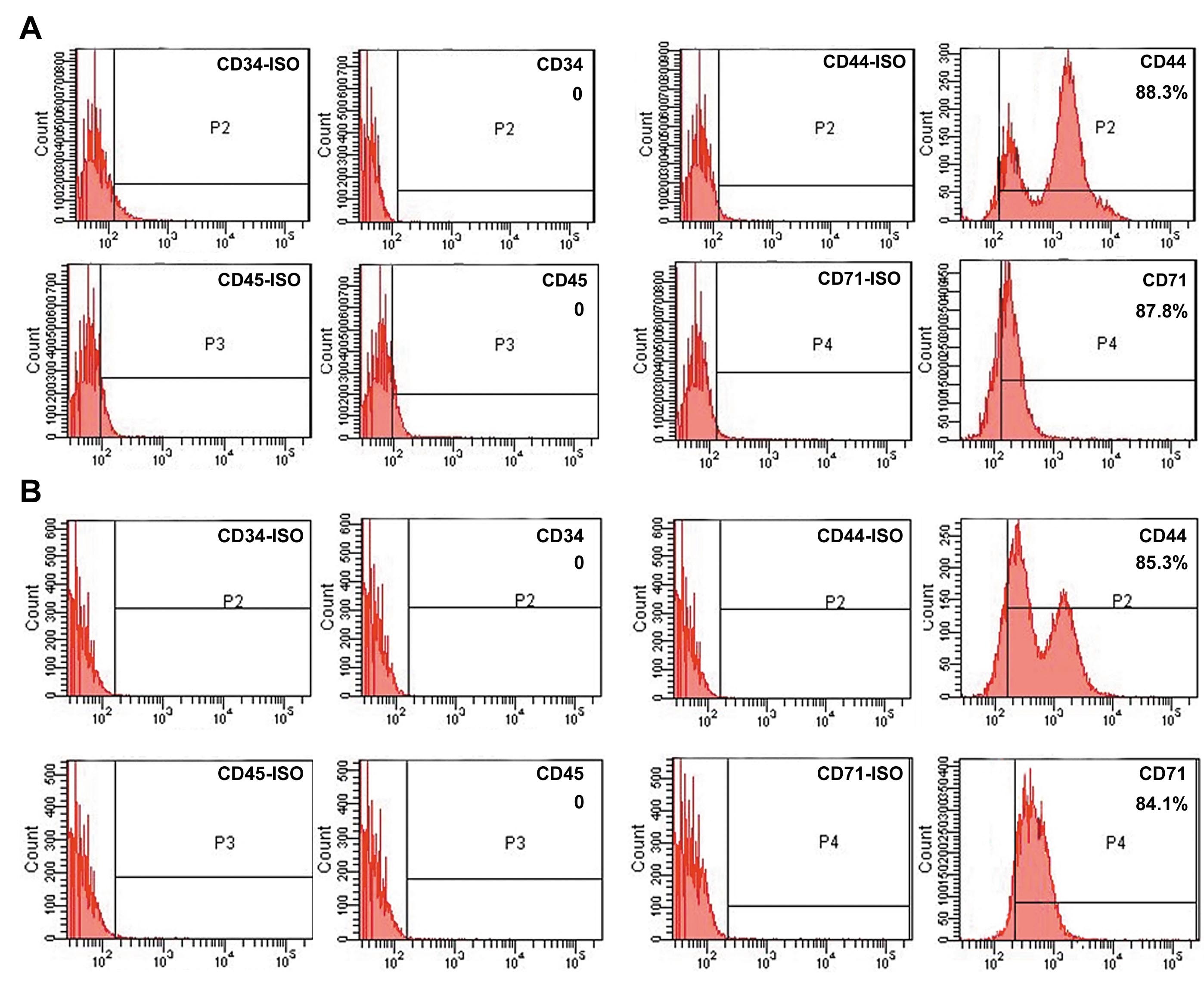

Supplement: Figure S1 — Flow cytometry analysis of phenotypic characteristics of rMSCs cultured under 21% O2 or 3% O2 for 3 days. (A) Phenotypic characteristics of rMSCs cultured under 21% O2. (B) Phenotypic characteristics of rMSCs cultured under 3% O2. (JPG) [file pone.0054296.s001.jpg]

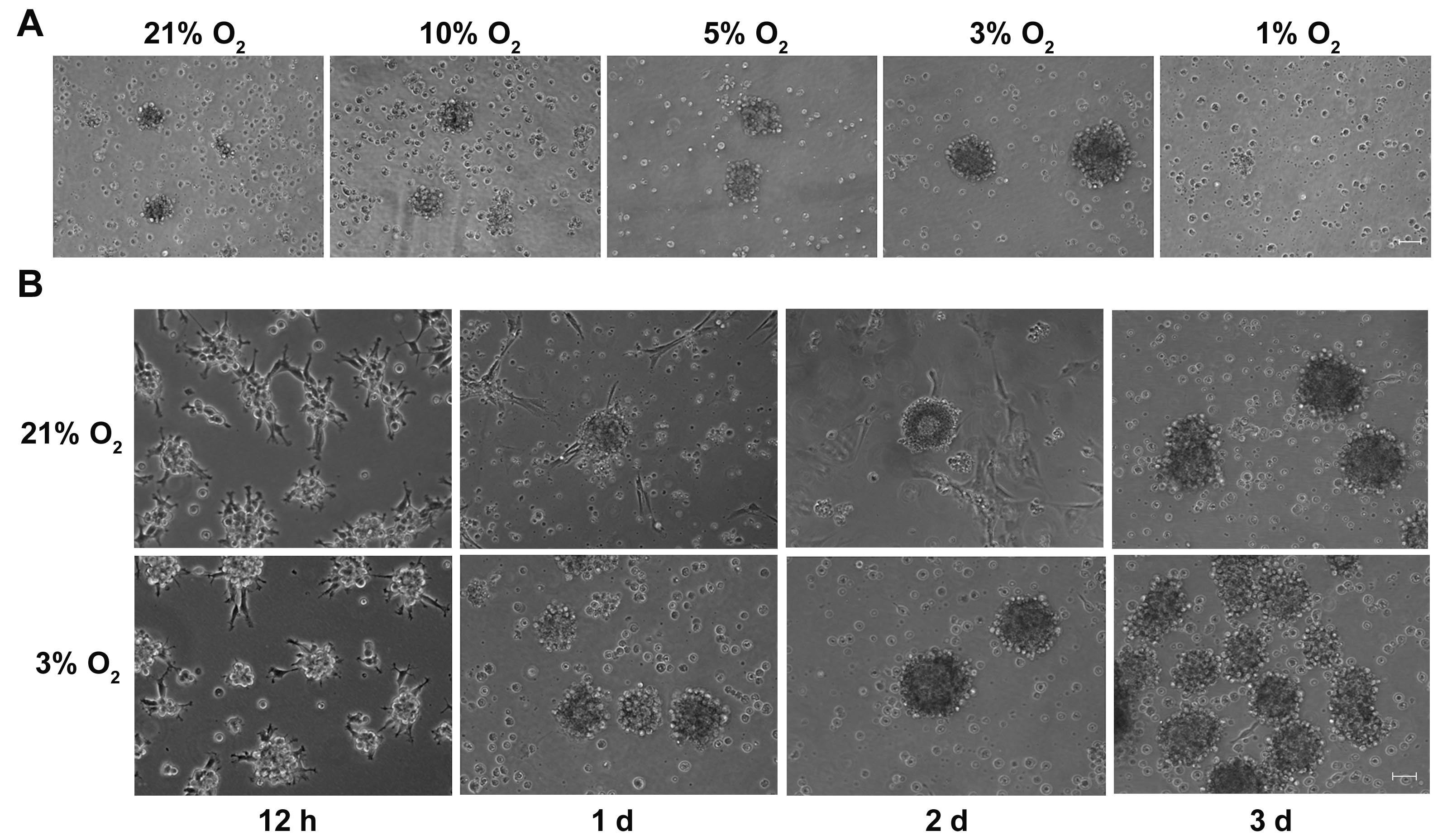

Supplement: Figure S2 — 3% O2 promotes the formation of neurosphere-like cell clusters from rMSCs. (A) Phase micrographs of rMSCs induced with neurosphere culture medium under different oxygen concentration at day 2. Scale bar = 100 µm. (B) Phase micrographs of rMSCs induced with neurosphere culture medium at different time points under 21% O2 or 3% O2. Scale bar = 100 µm. (JPG) [file pone.0054296.s002.jpg]

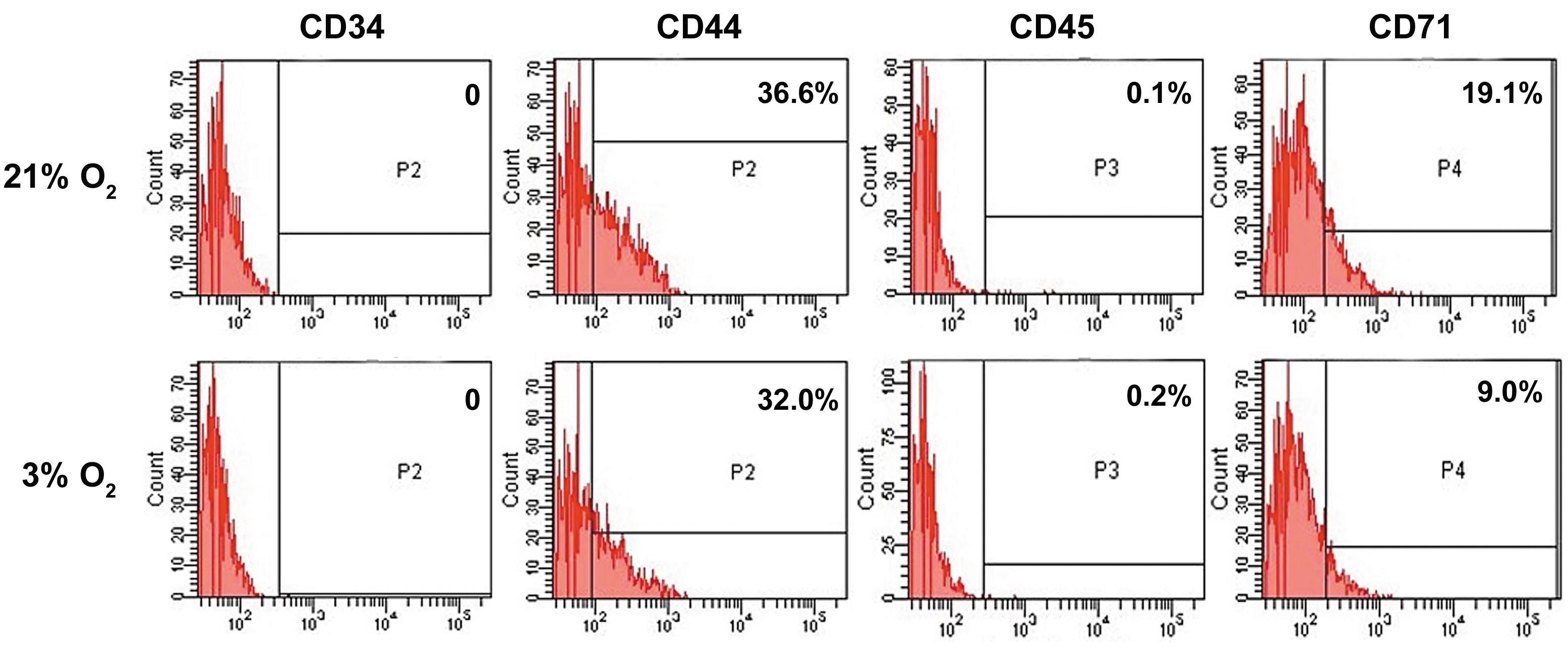

Supplement: Figure S3 — Flow cytometry analysis of phenotypic characteristics of rMSCs induced with neurosphere culture medium under 21% O2 or 3% O2 for 3 days. The expression of surface antigens CD44 and CD71 was remarkably decreased after induction, especially in 3% O2 group. (JPG) [file pone.0054296.s003.jpg]

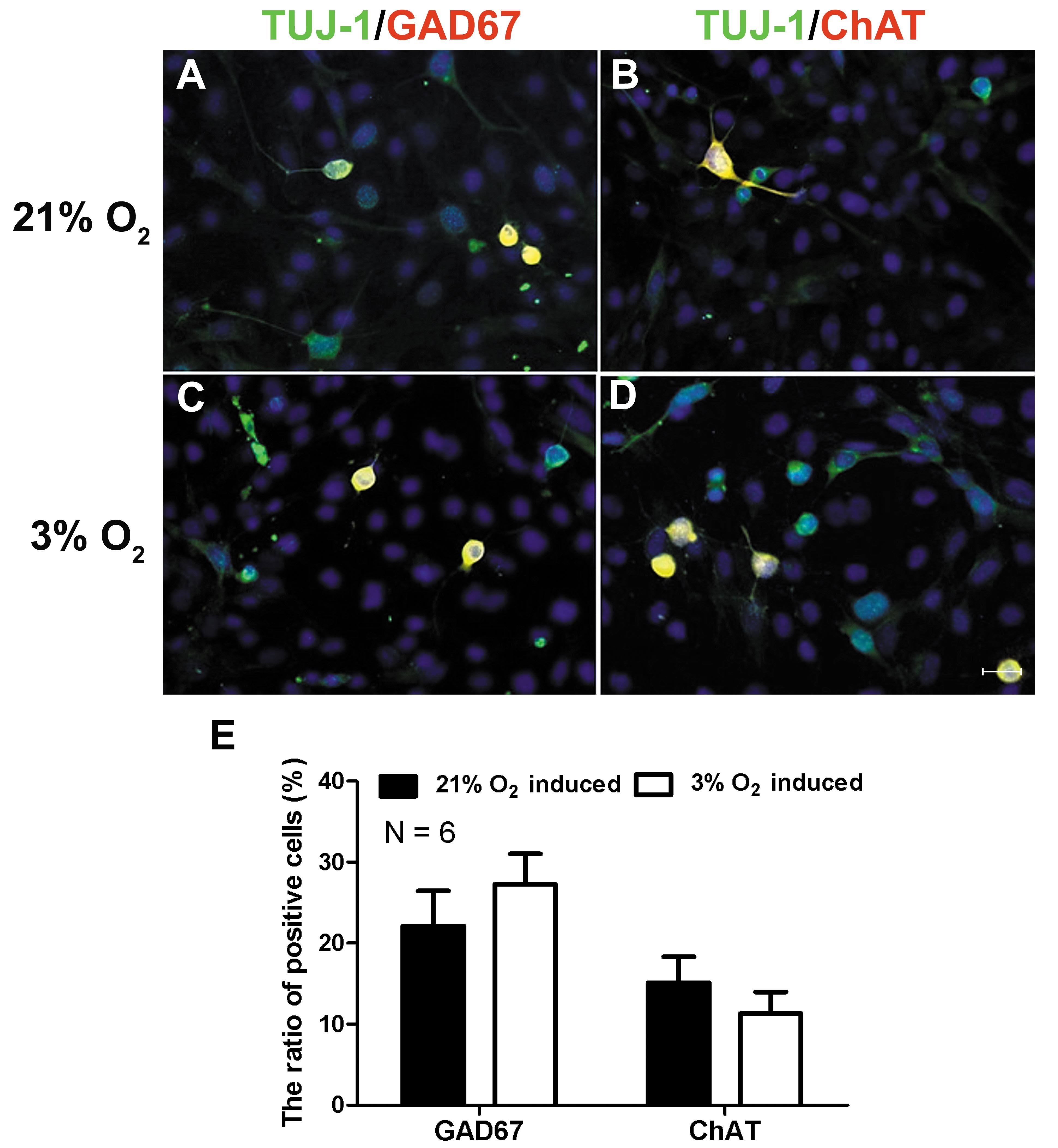

Supplement: Figure S4 — Double immunostaining of GAD67/TUJ-1 or ChAT/TUJ-1 in differentiated neurospheres. (A, C) Double immunostaining of GAD67 (red) and TUJ-1 (green). (B, D) Double immunostaining of ChAT (red) and TUJ-1 (green). (A, B) Double immunostaining for differentiated neurospheres generated under 21% O2. (C, D) Double immunostaining for differentiated neurospheres generated under 3% O2. (E) The percentage of GAD67+ or ChAT+ cells in TUJ-1+ cells in A–D. Scale bar = 20 µm for A–D. (JPG) [file pone.0054296.s004.jpg]

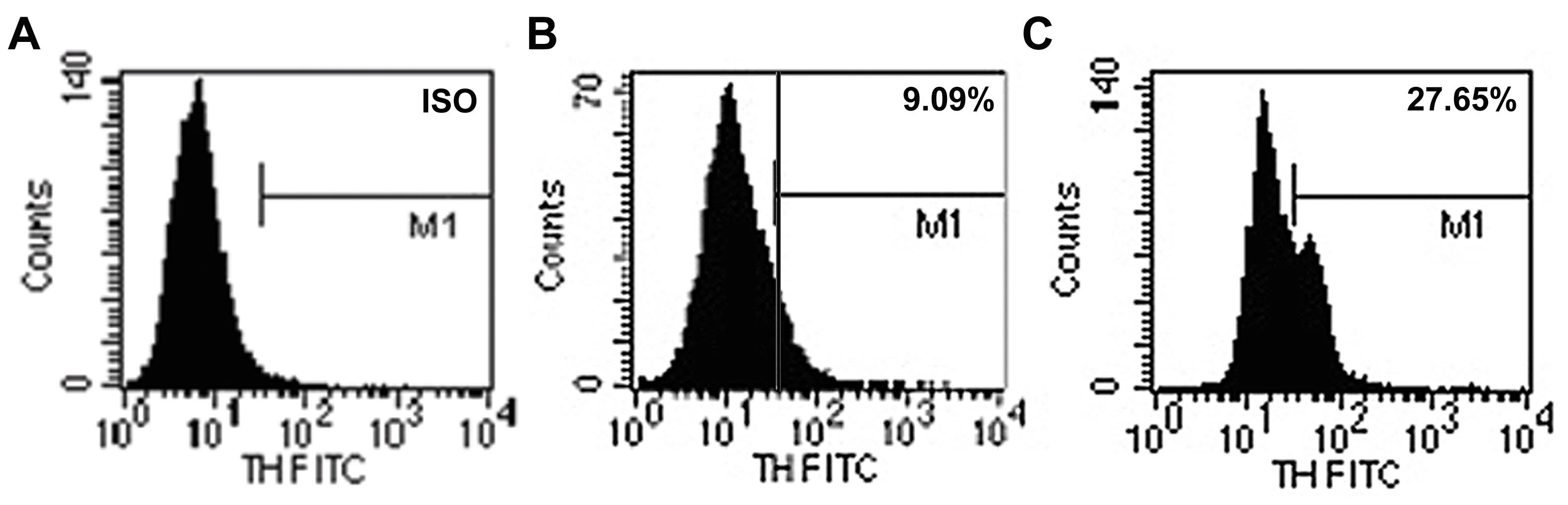

Supplement: Figure S5 — Flow cytometry analysis of TH+ cells in differentiated hMSCs. (A) Isotype control. (B) hMSCs differentiated under 21% O2. (C) hMSCs differentiated under 3% O2. (JPG) [file pone.0054296.s005.jpg]

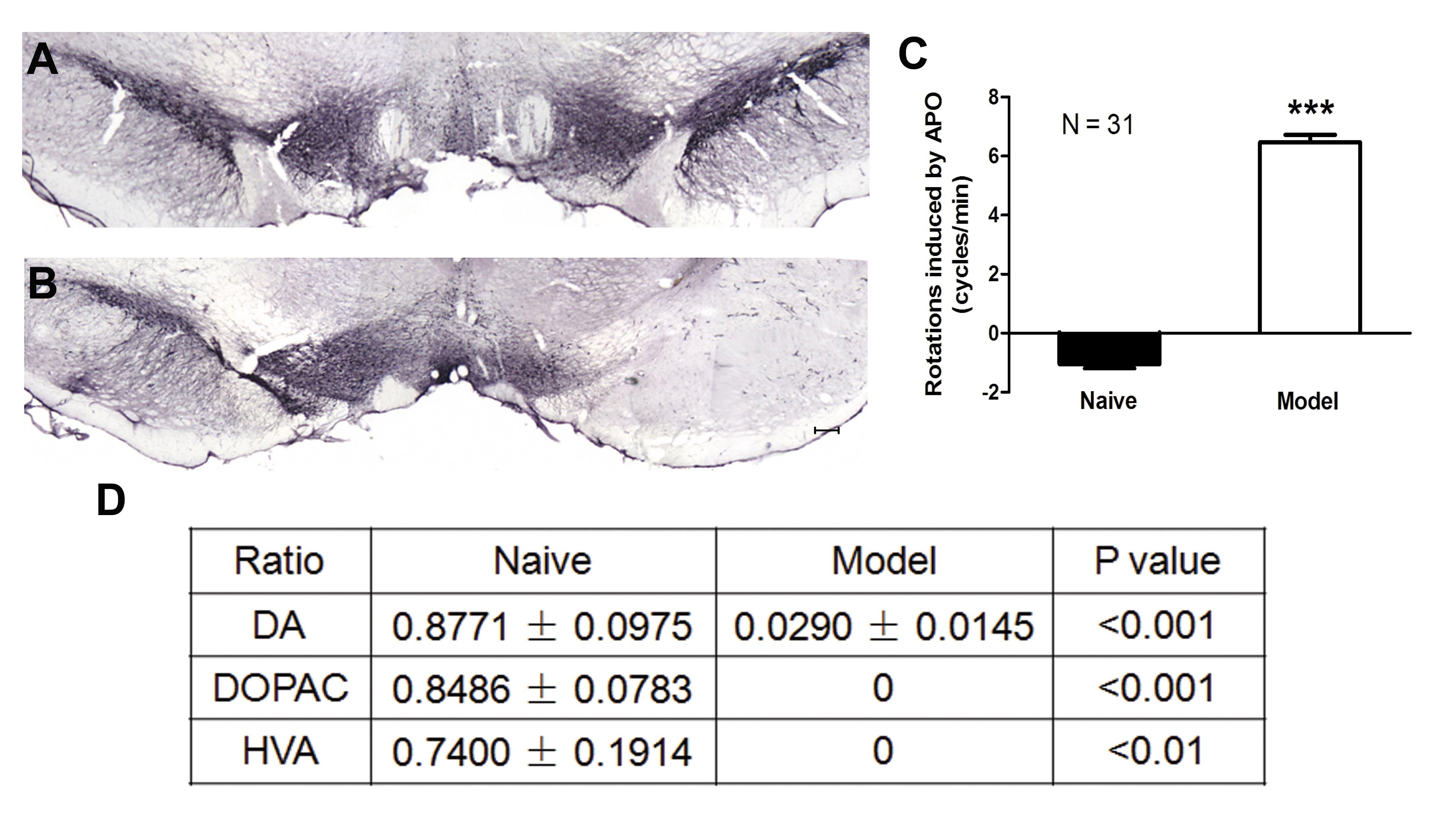

Supplement: Figure S6 — Establishment of parkinsonian rat model by unilateral injection with 6-OHDA into right medial forebrain bundle. (A, B) Immunohistochemistry staining of TH in the substantia nigra of brain sections from saline (A) or 6-OHDA (B) injected rats. (C) The number of apomorphine-induced rotation 4 weeks after 6-OHDA injection. (D) The relative striatal content of DA and its metabolites, DOPAC and HVA (lesioned side vs. unlesioned side). Data represent mean ± SEM, ***P<0.001. (JPG) [file pone.0054296.s006.jpg]

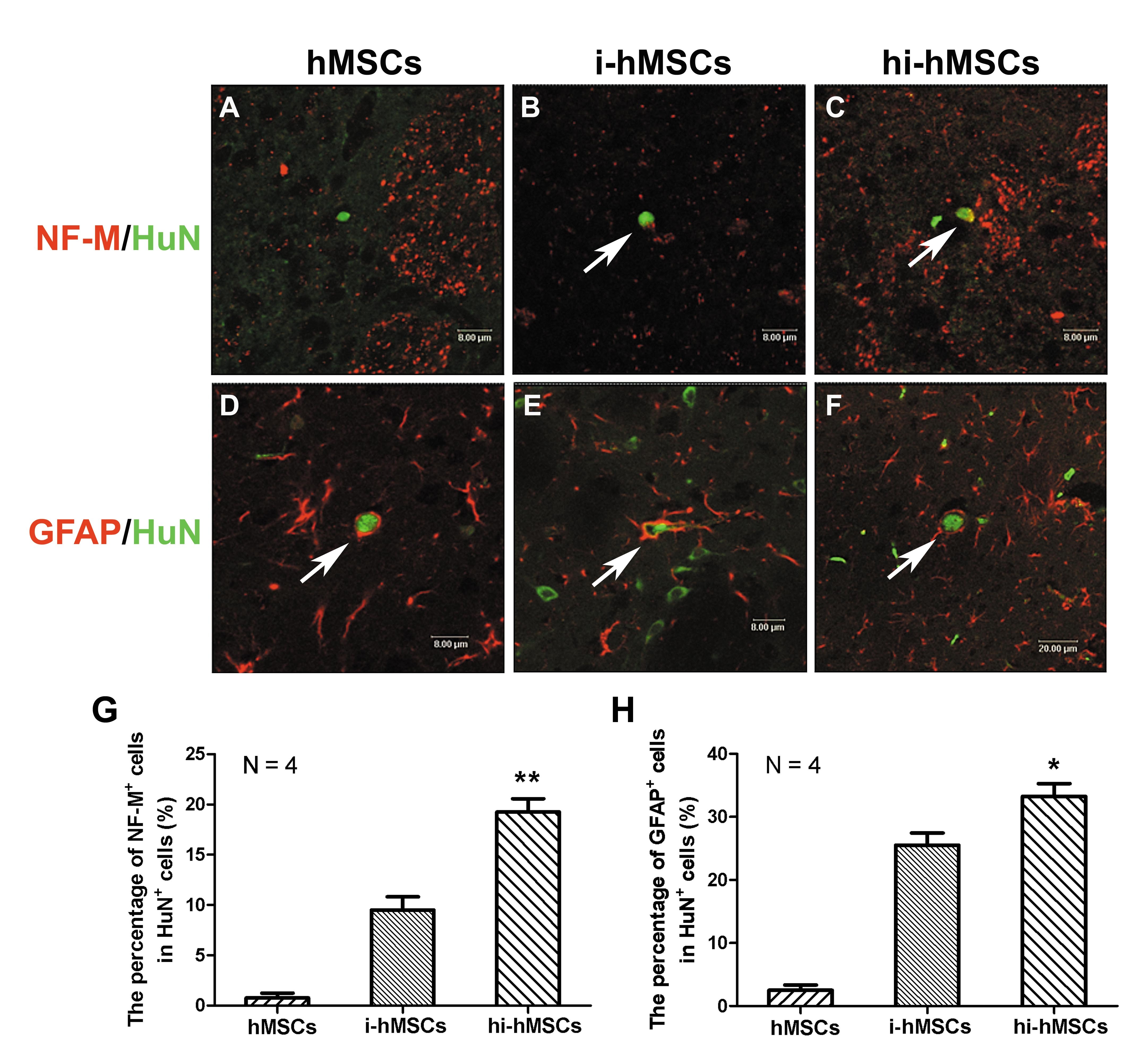

Supplement: Figure S7 — Double immunostaining of NF-M/HuN or GFAP/HuN in the striatum after transplantation. (A–C) Double immunostaining of NF-M (red) and HuN (green). (D–F) Double immunostaining of GFAP (red) and HuN (green). (A, D) PD model rats transplanted with hMSCs. (B, E) PD model rats transplanted with i-hMSCs. (C, F) PD model rats transplanted with hi-hMSCs. (G) The percentage of NF-M+ cells in HuN+ cells in A–F. (H) The percentage of GFAP+ cells in HuN+ cells in A–F. Scale bar = 8 µm for A–E. Scale bar = 20 µm for F. Data represent mean ± SEM, *P<0.05, **P<0.01. (JPG) [file pone.0054296.s007.jpg]
