# Supplementary material for: Hypoxia Promotes Dopaminergic Differentiation of Mesenchymal Stem Cells and Shows Benefits for Transplantation in a Rat Model of Parkinson’s Disease
Source: PLoS One. 2013 Jan 16;8(1):e54296. doi: 10.1371/journal.pone.0054296 (PMC3546985; doi:10.1371/journal.pone.0054296)
Supplement: Table S1 — Primer sequences for real-time PCR. (DOC) [file pone.0054296.s008.doc]

| Primers | Sequences |
| --- | --- |
| Nurr1-s | 5'-CTGCCCTGGCTATGGTCACAGA-3' |
| Nurr1-a | 5'-TTGGACAGGTAGTTGGGTCGGTT-3' |
| En1-s | 5'-GAACACAACCCCGCGATCCTAC-3' |
| En1-a | 5'-CAGAGGATGGACGGTCCGAGT-3' |
| En2-s | 5'-GCTGCAGAGGCTCAAAGCTGA-3' |
| En2-a | 5'-ATTTTGGCCCGCTTGTTCTGGAA-3' |
| Pitx3-s | 5'-GTCAGATGCAGGCACTCCACAC-3' |
| Pitx3-a | 5'-CTGCTTCTTCTTCAGGGAGCCAT-3' |
| Lmx1b-s | 5'-CAGCGGCTGCATGGAGAAGATC-3' |
| Lmx1b-a | 5'-GAGCACGAACTCATCACCCTTCC-3' |
| GAPDH-s | 5’-GTTACCAGGGCTGCCTTCTCTTG-3’ |
| GAPDH-a | 5’-CCTTGACTGTGCCGTTGAACTTG-3’ |
